# Spatial gradient consistency for unsupervised learning of hyperspectral demosaicking: application to surgical imaging

**DOI:** 10.1007/s11548-023-02865-7

**Published:** 2023-03-24

**Authors:** Peichao Li, Muhammad Asad, Conor Horgan, Oscar MacCormac, Jonathan Shapey, Tom Vercauteren

**Affiliations:** 1 School of Biomedical Engineering and Imaging Sciences, King’s College London, London, UK; 2 Department of Neurosurgery, King’s College Hospital NHS Foundation Trust, London, UK

**Keywords:** Hyperspectral imaging, Demosaicking, Unsupervised learning, Surgical imaging

## Abstract

**Purpose:**

Hyperspectral imaging has the potential to improve intraoperative decision making if tissue characterisation is performed in real-time and with high-resolution. Hyperspectral snapshot mosaic sensors offer a promising approach due to their fast acquisition speed and compact size. However, a demosaicking algorithm is required to fully recover the spatial and spectral information of the snapshot images. Most state-of-the-art demosaicking algorithms require ground-truth training data with paired snapshot and high-resolution hyperspectral images, but such imagery pairs with the exact same scene are physically impossible to acquire in intraoperative settings. In this work, we present a fully unsupervised hyperspectral image demosaicking algorithm which only requires exemplar snapshot images for training purposes.

**Methods:**

We regard hyperspectral demosaicking as an ill-posed linear inverse problem which we solve using a deep neural network. We take advantage of the spectral correlation occurring in natural scenes to design a novel inter spectral band regularisation term based on spatial gradient consistency. By combining our proposed term with standard regularisation techniques and exploiting a standard data fidelity term, we obtain an unsupervised loss function for training deep neural networks, which allows us to achieve real-time hyperspectral image demosaicking.

**Results:**

Quantitative results on hyperspetral image datasets show that our unsupervised demosaicking approach can achieve similar performance to its supervised counter-part, and significantly outperform linear demosaicking. A qualitative user study on real snapshot hyperspectral surgical images confirms the results from the quantitative analysis.

**Conclusion:**

Our results suggest that the proposed unsupervised algorithm can achieve promising hyperspectral demosaicking in real-time thus advancing the suitability of the modality for intraoperative use.

**Supplementary Information:**

The online version contains supplementary material available at 10.1007/s11548-023-02865-7.

## Introduction

Hyperspectral imaging (HSI) is a technique that captures and processes spectral data distributed across a large number of wavelengths. It provides a non-contact, non-ionising and non-invasive solution suitable for many medical applications [1–3]. HSI can provide information beyond what human vision can observe, such as tissue perfusion, oxygen saturation, and other diagnostic measurements [4]. Hence, it facilitates important medical tasks such as tissue differentiation and characterisation. Depending on the number of bands, hyperspectral imaging may also be called multispectral imaging, but in this work we will refer to hyperspectral imaging for consistency.

**Fig. 1:**
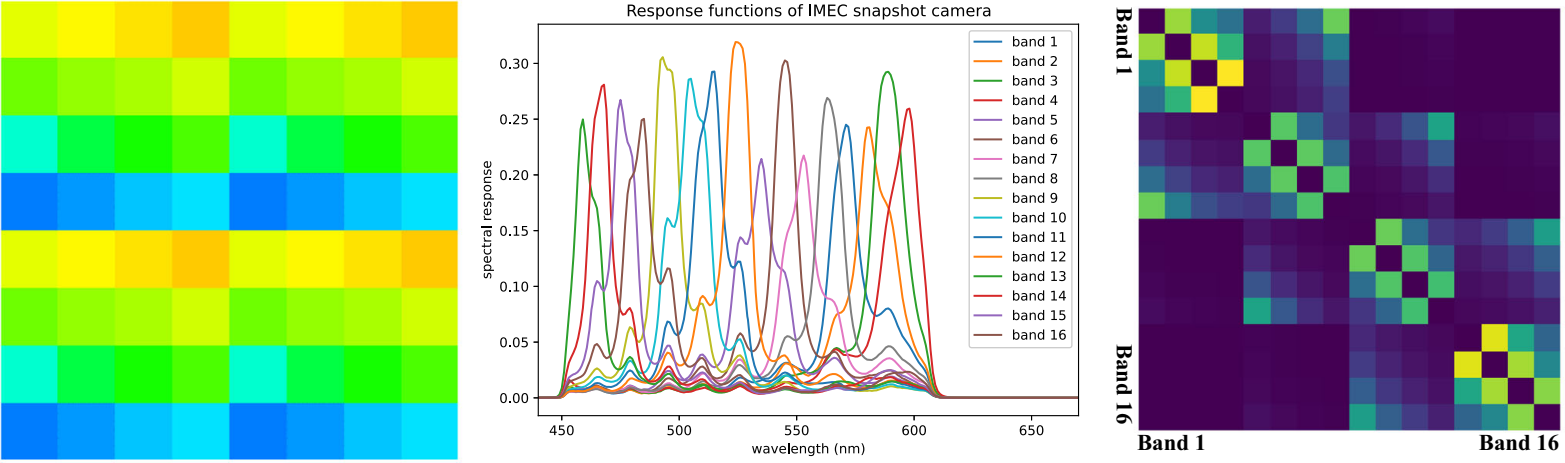
(Left) $4 \times 4$ MSFA for an IMEC snapshot camera, the colour of each pixel correlates to the perceived colour of a human observer. (Middle) Spectral responses of all 16 sensors on the MSFA of an IMEC snapshot camera. (Right) Wasserstein metric heatmap measuring distances between different spectral responses of the sensors on an IMEC snapshot camera

Snapshot hyperspectral imaging is a promising technique which can capture hyperspectral images in real-time. Snapshot mosaic cameras are a common type of snapshot hyperspectral camera which employ multi-spectral filter array (MSFA) to acquire multi-spectral data in a single exposure. In MSFA cameras the $n \times n$ sensor arrays are arranged in a repeating pattern similar to the $2 \times 2$ Bayer filter arrays on RGB cameras (Fig. 1, left) and are thus capable of obtaining a maximum of $n^2$ bands instantly. However, it achieves real-time multi-spectral data acquisition at the cost of reducing both spatial and spectral resolution. Efficient hyperspectral demosaicking algorithms are thus required to fully restore the spatial and spectral resolution from the snapshot images. More details on hyperspectral imaging techniques and snapshot mosaic imaging can be found in [5].

Traditionally, demosaicking algorithms were developed using interpolation-based methods or statistics-based techniques [6, 7], but these methods may still suffer from colour artifacts and blurriness. Recent deep-learning-based algorithms have been developed for efficient and accurate image super-resolution and demosaicking tasks. Deep neural networks such as SRCNN [8], EDSR [9] and RNAN [10] have demonstrated their performances on RGB image super-resolution tasks, and thus similar methods have been extended to process hyperspectral images [11, 12]. Arad et al. [13] introduced several state-of-the-art learning-based hyperspectral demosaicking algorithms of natural scenes in NTIRE 2022 Spectral Demosaicking Challenge. The leading contestants include Enhanced HAN [14], NLRAN [13] and Res2-Unet based methods [15]. Our previous work [5] also demonstrated the use of a synthetic surgical HSI dataset and deep-learning models for developing hyperspectral demosaicking algorithms suitable for intraoperative surgical guidance tasks.

However, most deep-learning-based demosaicking algorithms rely on a large number of high-resolution HSI data as the ground truth for model training. Publicly available medical hyperspectral datasets such as HELICoiD [16] and ODSI [17] involve large line-scan or spectral-scan HSI systems to obtain high-resolution hyperspectral data, and the acquisition speed is slow. Consequently, these imaging systems are not ideal for intraoperative use. Fortunately, [18] demonstrated that the acquisition of intraoperative snapshot mosaic images is less challenging as its compact imaging system can be seamlessly integrated into a standard surgical workflow.

This paper presents an unsupervised-learning-based HSI demosaicking algorithm which uses only snapshot mosaic images and does not require corresponding high-resolution images for training. A demosaicking loss function is proposed based on a novel spatial gradient consistency regularisation technique combined with traditional regularisation methods including Tikhonov regularisation and total variation. The proposed algorithm has been tested with 3 different deep neural networks on 3 different datasets. Quantitative measures have been performed to compare the unsupervised algorithm against linear demosaicking and supervised training, and a qualitative user study was conducted to validate the proposed algorithm on a medical HSI dataset.

## Materials and methods

### Demosaicking as an ill-posed linear inverse problem

**Problem formulation** Hyperspectral image demosaicking involves recovering the fully sampled hyperspectral image $I \in \mathbb {R}^{X \times Y \times C}$ from a snapshot image $I^s \in \mathbb {R}^{X \times Y}$, where *X* and *Y* are the spatial dimensions and *C* is the number of spectral bands. The relationship between *I* and $I^s$ can be expressed through a linear degradation operator $\mathcal {D}$:

1 $$\begin{aligned} I^s=\mathcal {D}(I) \end{aligned}$$

For a typical MSFA arrangement as shown in Fig. 1 (left), $\mathcal {D}$ can be simply expressed as a selection matrix containing only 0 and 1, thereby mapping the pixel values of $I^s$ from *I*. In other words, for each spatial location (*x*, *y*), there is a single corresponding spectral band $c_{x,y}$ such that $I^s(x,y) = I(x,y,c_{x,y})$ The inverse problem corresponding to (1) is ill-posed because of the highly ill-conditioned selection operator $\mathcal {D}$. Therefore, appropriate regularisation is required. A classical inverse problem approach would aim at solving for

2 $$\begin{aligned} \hat{I} = \arg \min _I \big [ \mathcal {L}(I^s,\mathcal {D}(I)) + \lambda \mathcal {R}(I) \big ] \end{aligned}$$

where $\mathcal {L}(I^s,\mathcal {D}(I))$ is the data fidelity term that measures the differences between the known snapshot image $I^s$ and the subsampling of the unknown fully-sampled hyperspectral image *I*. $\mathcal {R}$ represents the regularisation terms. $\lambda$ is the regularisation factor that determines the trade-off between the data fidelity and regularisation.

Translating this into an unsupervised machine learning setting, we now seek to optimise for the parameters $\theta$ of a deep neural network $f_{\theta }$ mapping a snapshot mosaic input $I^s$ to a fully-sampled hyperspectral image $f_{\theta }(I^s)$:

3 $$\begin{aligned} \hat{\theta } = \arg \min _\theta \mathbb {E}_{I^s} \big [ \mathcal {L}(I^s,\mathcal {D}(f_{\theta }(I^s))) + \lambda \mathcal {R}(f_{\theta }(I^s)) \big ] \end{aligned}$$

where the expectation $\mathbb {E}_{I^s}$ is to be considered as being taken over an empirical distribution defined by a training set of snapshot mosaic images (with no need for ground truth).

**Spatial gradient consistency regularisation** Regularisation terms in (3) aim at incorporating prior information about the problem being solved. In our case, all spectral bands are imaging the same physical scene. We also observe that the spectrum of natural objects and biological tissues present with specific characteristics such as continuity and smoothness. Additionally, the response functions corresponding to the different spectral bands as shown in Fig. 1 (middle) shares significant spectral overlap. It is thus expected that our spectral bands will exhibit substantial correlation. Inter-spectral band correlation was notably demonstrated empirically for RGB images in [19]. However, while correlation is expected, assuming a simple linear relationship would make for too crude an approximation.

Here, inspired by image similarity metrics that exploit image gradients for multimodal image registration where non-trivial correlation across the imaging modalities is expected [20], we propose to promote correlation between the spatial gradients of the individual spectral bands in our reconstructions. Let $c_1$ and $c_2$ be the indices of two spectral bands of interest, with $I^{c}=I(\cdot ,\cdot ,c)$, and $c\in (c_1,c_2)$ the corresponding spectral band images. For simplicity, we make use of forward differences to compute spatial gradients: $\nabla _x I^c(x,y)=I^c(x+1,y)-I^c(x,y)$ and $\nabla _y I^c(x,y)=I^c(x,y+1)-I^c(x,y)$. We propose to consider the correlation coefficient between the spatial gradients as a regularisation:

4 $$\begin{aligned} \mathcal {R}^{c_1,c_2}_{\rho }(I) = - \rho (\nabla _x I^{c_1}, \nabla _x I^{c_2}) - \rho (\nabla _y I^{c_1}, \nabla _y I^{c_2}) \end{aligned}$$

Given *C* spectral bands, $C^2$ pairwise comparisons are possible. However, the strength of the correlation is not expected to be the same for all pairs of bands. Indeed, two bands with close spectral peaks should lead to higher correlation than two bands with further peaks. Given the complex structure of the spectral response functions shown in Fig. 1 (middle), we propose to weight the contribution of each pair of spectral band according to the Wasserstein distance $W_{c_1,c_2}$ between the spectral response functions of the two bands:

5 $$\begin{aligned} \mathcal {R}_{\rho }(I) = \sum _{c_1 \ne c_2} e^{-\frac{W_{c_1,c_2}}{\tau }} ~ \mathcal {R}^{c_1,c_2}_{\rho }(I) \end{aligned}$$

where the negative exponential mapping with temperature scaling $\tau$ allows to control the relative importance of each pair. The exponential Wasserstein distance gives an indication of how closely the spectral responses of the two bands might be correlated, as shown in the heatmap in Fig. 1 (right), where lighter colour means the two spectral bands are closer. By strengthening the correlation between the spatial gradient maps of different spectral bands we expect to enhance the sharp edges and contours.

**Other regularisation terms** Tikhonov regularisation is a common method for ill-conditioned problems. It can be characterised as:

6 $$\begin{aligned} \mathcal {R}_{\text {Tik}}(I)=\Vert \varvec{\Gamma } \cdot I\Vert _2^2 \end{aligned}$$

Here, we choose to use the Laplacian matrix as the Tikhonov matrix $\varvec{\Gamma }$ to deal with potential high-frequency artifacts introduced during the super-resolution process. While Tikhonov regularisation can effectively eliminate undesirable outliers and led to smooth images, it also has the potential risk of applying too much smoothness and erasing all sharp edges and contours, which is harmful for recovering details in the images.

Total variation is another term which is able to preserve edges while regularising solutions of the inverse problem:

7 $$\begin{aligned} \mathcal {R}_{\text {TV}}(I) = \Vert \nabla _x I\Vert _1 + \Vert \nabla _y I\Vert _1 \end{aligned}$$

By combining our proposed spatial gradient consistency term with Tikhonov and total variation regularisation, we obtain the regularisation term $\mathcal {R}$ in (2) using $\lambda _{\text {Tik}}$, $\lambda _{\text {TV}}$ and $\lambda _{\rho }$ as weighting factors for individual terms:

8 $$\begin{aligned} \mathcal {R}(I) = \lambda _{\text {Tik}} \mathcal {R}_{\text {Tik}}(I) + \lambda _{\text {TV}} \mathcal {R}_{\text {TV}}(I) + \lambda _{\rho } \mathcal {R}_{\rho }(I) \end{aligned}$$

### Image demosaicking pipeline

**Fig. 2:**
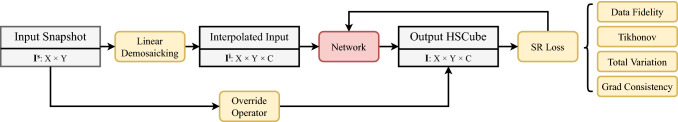
The pipeline of the proposed unsupervised demosaicking algorithm

Figure 2 depicts the general pipeline of our proposed algorithm using deep neural networks for hyperspectral image demosaicking problems. It starts from the input snapshot mosaic images where bilinear interpolation-based demosaicking can be applied to recover the spatial and spectral dimension of the images. The linearly interpolated images serve as the input of the network to generate refined demosaicking results. Most deep neural networks for image super-resolution or demosaicking can be integrated into this pipeline, such as U-Net [21], EDSR [9] and Res2-Unet [15].

Aside from the network, given that the measured pixels in the original snapshot $I^s$ should be equal to the corresponding pixels in the demosaicked hypercube *I*, we propose to include an overriding operator which applies the pixel values from $I^s$ to their corresponding position in *I*. This forces the data fidelity term $\mathcal {L}$ in (2) to be always 0 irrespective of the metric we choose. Based on the output images from the network with the overridden snapshot pixels, the Tikhonov regularisation, total variation and the spatial gradient consistency regularisation terms are calculated and minimised using gradient descent, and the parameters in the networks are updated.

### Source datasets

To experiment the proposed demosaicking algorithm, three hyperspectral imaging datasets are used in this work, which will be presented in this section.

**HELICoiD** Fabelo et al. [16] presented a publicly available in-vivo hyperspectral human brain image dataset within the European project HELICoiD (HypErspectraL Imaging Cancer Detection). The hyperspectral images in this dataset were acquired using a line-scan hyperspectral camera system capable of capturing high spectral-resolution hypercubes during neurosurgical operations. The dataset contains 36 images in the Visual and Near Infrared (VNIR) range from 400nm to 1000nm. We applied the same method described in Li et al. [5] to perform white balancing, and then simulated snapshot mosaic images and their corresponding high-resolution demosaicked hypercubes using spectral response functions of a real hyperspectral snapshot camera.

**ARAD_1K** With the NTIRE 2022 Spectral Demosaicking Challenge, Arad et al. [13] provided 1000 hyperspectral images of natural scenes with 16 spectral bands ranging from 400 nm to 1000 nm. The snapshot images were simulated following a $4 \times 4$ MSFA pattern. There were 950 hyperspectral images for training, where the simulated snapshot images and their corresponding ground truth images were both provided. The other 50 images were for testing, but the ground truth was not publicly available, so we separated 50 images out from the 950 training set for testing.

**NeuroHSI** NeuroHSI is an actively running, NIHR funded, single centre prospective observational study assessing the intra-operative capabilities of a $4 \times 4$, 16 band visible range snapshot mosaic camera (IMEC CMV2K-SSM4X4-VIS) to differentiate between pathological tissue and healthy brain tissue, as well as to evaluate custom made algorithms capable of correlating information from specific bands to tissue oxygenation measurements. Phase one of this study has now been completed and video hyperspectral data from two brain metastases, two gliomas (WHO grades 2–4), one meningiomas, one vestibular schwannoma, one cerebral aneurysm and one cerebral arteriovenous malformation has been collected. 150 snapshot images with minor motion blur or out-of-focus blur were manually selected from the video data of the 8 patients, where 90 images from 4 patients are reserved for training, 30 images from 2 patients reserved for validation and 30 from the remaining 2 patients for testing.

**Table 1:** Comparison of demosaicking accuracy between linear demosaicking and different networks with supervised and unsupervised training setup on HELICoiD and ARAD_1K datasets

Dataset	Method	Network	SSIM	PSNR	SAM
HELICoiD	Linear	–	$0.969 \pm 0.011$	$32.5 \pm 2.48$	$0.123 \pm 0.051$
	Supervised	UNet	$0.991 \pm 0.005$	$38.7 \pm 3.06$	$0.057 \pm 0.025$
		EDSR	$0.994 \pm 0.004$	$42.3 \pm 3.70$	$0.035 \pm 0.013$
		Res2-Unet	$\mathbf {0.995 \pm 0.004}$	$\mathbf {42.9 \pm 3.99}$	$\mathbf {0.032 \pm 0.012}$
	Unsupervised	UNet	$0.991 \pm 0.004$	$38.6 \pm 3.43$	$0.057 \pm 0.024$
		EDSR	$\mathbf {0.993 \pm 0.004}$	$\mathbf {39.9 \pm 3.70}$	$\mathbf {0.046 \pm 0.019}$
		Res2-Unet	$0.992 \pm 0.005$	$39.7 \pm 3.66$	$0.051 \pm 0.019$
ARAD_1K	Linear	–	$0.956 \pm 0.039$	$33.4 \pm 4.16$	$0.055 \pm 0.022$
	Supervised	UNet	$0.978 \pm 0.022$	$38.4 \pm 3.91$	$0.041 \pm 0.016$
		EDSR	$0.993 \pm 0.007$	$43.1 \pm 3.74$	$0.025 \pm 0.009$
		Res2-Unet	$\mathbf {0.998 \pm 0.002}$	$\mathbf {48.7 \pm 3.88}$	$\mathbf {0.015 \pm 0.006}$
	Unsupervised	UNet	$0.977 \pm 0.020$	$38.1 \pm 3.62$	$0.041 \pm 0.015$
		EDSR	$\mathbf {0.989 \pm 0.013}$	$41.0 \pm 4.07$	$0.031 \pm 0.013$
		Res2-Unet	$0.988 \pm 0.017$	$\mathbf {41.3 \pm 4.42}$	$\mathbf {0.031 \pm 0.012}$

The results with the highest demosaicking accuracy among different networks are highlighted in bold

### Implementation details

Our proposed algorithm was implemented with PyTorch and tested on all three datasets described in Sect. “Source datasets”. For the HELICoiD dataset, synthetic snapshot images and their corresponding high-resolution hypercubes were simulated using sensor information from the snapshot camera IMEC CMV2K-SSM4X4-VIS. The dataset was divided into 3 groups: 24 images acquired from 15 different patients as the training set, 6 images from 4 patients as the validation set, and the remaining 6 images from 3 patients as the test set. For the ARAD_1K dataset, the original raw snapshot data were simulated with an unknown exposure setting. Recovering such an unknown exposure is not the primary focus for our experiment. Therefore, new snapshot images were simulated using the ground truth hypercubes and the MSFA simulation algorithm provided by the organiser. The dataset was also divided into 3 groups: 720 images for training, 180 for validation and 50 for testing.

As both the HELICoiD and ARAD_1K datasets have high-resolution hypercubes as ground truths, the U-Net, EDSR and Res2-Unet models were trained in both a supervised and an unsupervised manner. For supervised training, the models were all trained using the Mean Relative Absolute Error (MRAE) Loss as described in Song et al. [15]. For unsupervised training, the regularisation terms described in (8) were used as the loss function, and the models were trained with only the simulated snapshot images as inputs. The regularisation factors in (8) were set to $\lambda _{\text {Tik}}=1$, $\lambda _{\text {TV}}=10^{-3}$ and $\lambda _{\rho }=1$ respectively, and the temperature scaling $\tau$ in (5) was set to 0.1. Details on the parameter selection and the ablation study can be found in the supplementary material. Random flipping and rotation were not performed because they can disrupt the MSFA pattern of the snapshot images. Therefore, random divisible spatial cropping were performed where the position and size of the crop were all divisible by the size of the mosaic. The network models were trained using the Adam optimiser with $\beta _1=0.5$ and $\beta _2=0.99$ and a batch size of 4. The initial learning rate was set to $1 \times 10^{-4}$. Results were quantitatively evaluated based on 3 metrics, including Structural Similarity (SSIM), Peak Signal-to-Noise Ratio (PSNR) and Spectral Angle Mapper (SAM) [22].

The 150 image frames selected from the NeuroHSI video dataset were all acquired from an IMEC CMV2K-SSM4X4-VIS camera, and there are no ground truth high-resolution hypercubes, so the experiment only involves unsupervised training. Ninety snapshot image frames from 4 patients were used for training, and 30 images from 2 patients for both validation and testing. Res2-Unet was adopted for the proposed algorithm, and the parameters used for training on NeuroHSI dataset remains the same as the HELICoiD and ARAD_1K dataset. The results were evaluated qualitatively by a user study which will be described in Sect. “Qualitative evaluation and user study”.

**Fig. 3:**
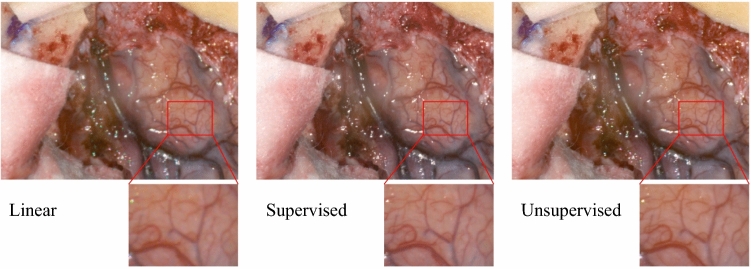
Comparison between different demosaicking methods on an example NeuroHSI test image. The reconstructed sRGB images are converted from the demosaicked hyperspectral data following the method described in [5]

## Results

### Quantitative evaluation

The quantitative results of the demosaicked hypercubes on both HELICoiD and ARAD_1K datasets are shown in Table 1. Paired T-test was performed to compare against the performance of two demosaicking methods. For both datasets, the supervised training of Res2-Unet achieved the highest demosaicking accuracy. The supervised EDSR results did not show statistical differences compared to Res2-Unet at a significant level of 0.05 on the HELICoiD dataset, with *p*-values of 0.35, 0.34 and 0.30 for SSIM, PSNR and SAM respectively. However, on the ARAD_1K dataset the *p*-values of $<10^{-5}$ for all 3 metrics indicates that Res2-Unet outperforms EDSR significantly.

The demosaicking results of the proposed unsupervised method on Res2-Unet are significantly lower than the supervised method with *p*-values of 0.040, 0.016, 0.007 on the 3 metrics on HELICoiD dataset, and *p*-values of close to 0 on ARAD_1K dataset, showing that our proposed method cannot match state-of-the-art supervised demosaicking methods when ground truths are provided. However, when comparing supervised and unsupervised EDSR results, the *p*-values of 0.17, 0.06 and 0.07 on the HELICoiD dataset indicates that our proposed method can still reach similar performance as a supervised method. On the ARAD_1K dataset, although the unsupervised EDSR performs significantly lower than supervised EDSR with p-values of 0.02, 0.0001 and 0.0005, it still outperforms the supervised U-Net significantly with *p*-values of $<10^{-5}$ for all 3 metrics. In both datasets, all supervised and unsupervised results significantly outperform linear demosaicking with *p*-values close to 0.

The speed of our proposed demosaicking algorithm depends on the choice of network. For a single image of size $512 \times 480$ from the ARAD_1K dataset, the inference times for UNet, EDSR and Res2-Unet are around 0.009 s, 0.006 s and 0.010 s respectively with NVIDIA RTX 3080 Ti. This demonstrates that when combining a suitable neural network and computing hardware, our proposed algorithm can achieve high quality hyperspectral demosaicking in real-time.

### Qualitative evaluation and user study

As there is no ground truth data for the NeuroHSI dataset, a qualitative user study was conducted to evaluate the demosaicked results of the NeuroHSI dataset. The user study was conducted using forced-choice pairwise comparison [23]. Figure 3 illustrates the pseudo-sRGB reconstructions of an example NeuroHSI patient image tested using three methods: linear demosaicking (L), supervised Res2-Unet model trained from HELICoiD dataset (SL) and the unsupervised Res2-Unet model trained from NeuroHSI training set (UL). 30 test images were included in the user study, each tested with the three methods (L, SL, UL). There are thus 90 questions in total, each containing two images of the same scene with 2 different demosaicking methods. These questions were divided into 3 separate surveys, each containing 30 questions. Participants were randomly assigned to answer one of 3 surveys and asked to choose the image with better quality for each question (pair of images) without any knowledge of which demosaicking method was used. The participants of this survey were all neurosurgical experts with 2–15 years of experience. We received 12 responses in total that are summarised in Table 2. We applied the Bradley-Terry model [24] to rank the demosaicking methods, which gives the estimated preference scale of $\pi =(0.050, 0.445, 0.505)$ for L, SL and UL respectively. This indicates that the experts considered the images recovered from our proposed demosaicking method to have similar quality as the images from a supervised model, with the baseline linear demosaicking the least favourable method. More details can be found in the supplementary material.

**Table 2:** Number of votes received for each demosaicking method in all pairwise comparisons in the image quality assessment survey

Linear	Supervised	Linear	Unsupervised	Supervised	Unsupervised
13	107	10	110	57	63

## Conclusion

In this work, we have presented a novel unsupervised approach for medical hyperspectral image demosaicking. The proposed algorithm does not rely on high-resolution medical hyperspectral data which are hard to acquire in a surgical environment, but instead only snapshot mosaic images are required, which are much easier to capture. The combination of Tikhonov regularisation, total variation and spectral correlation regularisation has been adopted for unsupervised network training, and the results were tested both quantitatively and qualitatively, showing convincing results over basic linear demosaicking, and comparable results against supervised demosaicking methods, thus proving its capability for real-time intraoperative surgical application.

## Supplementary Information

Below is the link to the electronic supplementary material.

Supplementary file 1 (pdf 7373 KB)
