# Supplementary material for: Spatial gradient consistency for unsupervised learning of hyperspectral demosaicking: application to surgical imaging
Source: Int J Comput Assist Radiol Surg. Author manuscript; Available in PMC 2023 Jun 26. (PMC10284955; doi:10.1007/s11548-023-02865-7)
Supplement: Electronic supplementary material [file EMS174845-supplement-Electronic_supplementary_material.pdf]

Supplementary material
Spatial gradient consistency for unsupervised learning of hyperspectral demosaicking: application to surgical imaging

Peichao Li[1*], Muhammad Asad[1], Conor Horgan[1], Oscar MacCormac[1,2], Jonathan Shapey[1,2] and Tom Vercauteren[1]

[1]School of Biomedical Engineering & Imaging Sciences, King's College London, London, UK.
[2] Department of Neurosurgery, King's College Hospital NHS Foundation Trust, London, UK.

*Corresponding author(s). E-mail(s): peichao.2.li@kcl.ac.uk;

1 Evaluation Metrics

The evaluation metrics used in our quantitative analysis of the demosaicking results include Structural Similarity index (SSIM), Peak Signal-to-Noise Ratio (PSNR) and Spectral Angle Mapper (SAM) [?]. Given the ground truth hyperspectral image $I \in \mathbb{R}^{X \times Y \times C}$ and the demosaicked hyperspectral image $\hat{I} \in \mathbb{R}^{X \times Y \times C}$, the SSIM can be calculated based on the luminance term $l(I, \hat{I})$, the contrast term $c(I, \hat{I})$ and the structural term $s(I, \hat{I})$:

$$l(I, \hat{I}) = \frac{2\mu_I \mu_{\hat{I}} + C_1}{\mu_I^2 + \mu_{\hat{I}}^2 + C_1} \tag{1}$$

$$c(I, \hat{I}) = \frac{2\sigma_I \sigma_{\hat{I}} + C_2}{\sigma_I^2 + \sigma_{\hat{I}}^2 + C_2} \tag{2}$$

$$s(I, \hat{I}) = \frac{\sigma_{I\hat{I}} + C_3}{\sigma_I \sigma_{\hat{I}} + C_3} \tag{3}$$

where μ_I, $\mu_{\hat{I}}$, σ_I, $\sigma_{\hat{I}}$ and $\sigma_{I\hat{I}}$ represent the means, standard deviations and the cross covariance of the hyperspectral images I and $\hat{I}$. C_1, C_2 and C_3 are

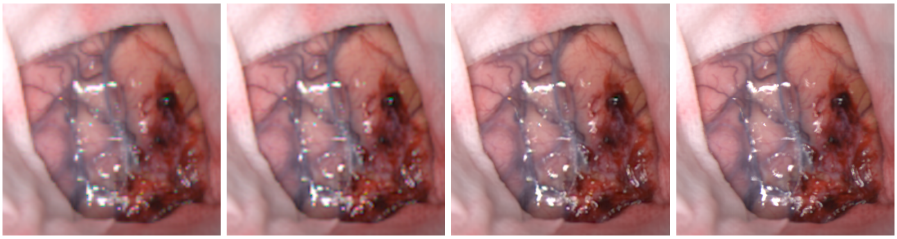

Fig. 1 The effect of the proposed gradient consistency regularisation term on the demosaicked image.

constants to ensure stability when the denominator becomes 0. SSIM is then given by:

$$SSIM(I,\hat{I}) = [l(I,\hat{I})]^{\alpha} \cdot [c(I,\hat{I})]^{\beta} \cdot [s(I,\hat{I})]^{\gamma} \tag{4}$$

To simplify the expression, by default $\alpha = \beta = \gamma = 1$, and $C_3 = 0.5C_2$. Hence, SSIM can be simplified as:

$$SSIM(I,\hat{I}) = \frac{(2\mu_I\mu_{\hat{I}} + C_1)(2\sigma_{I\hat{I}} + C_2)}{(\mu_I^2 + \mu_{\hat{I}}^2 + C_1)(\sigma_I^2 + \sigma_{\hat{I}}^2 + C_2)} \tag{5}$$

PSNR is defined as:

$$PSNR = 10\log_{10}(\frac{MAX_I^2}{MSE}) \tag{6}$$

where MAX_I^2 is the maximum pixel value in I, and MSE is the mean-squared error which is defined as:

$$MSE = \frac{1}{XYC}\sum_{x=1}^{X}\sum_{y=1}^{Y}\sum_{c=1}^{C}(I(x,y,c) - \hat{I}(x,y,c))^2 \tag{7}$$

Finally, SAM can be calculated by:

$$SAM = \frac{1}{XY}\sum_{x=1}^{X}\sum_{y=1}^{Y}\arccos(\frac{\sum_{c=1}^{C} I(x,y,c)\cdot\hat{I}(x,y,c)}{\sqrt{\sum_{c=1}^{C} I(x,y,c)^2}\sqrt{\sum_{c=1}^{C}\hat{I}(x,y,c)^2}}) \tag{8}$$

2 Ablation Study

Figure 1 shows an example test image from HELICoiD [?] dataset illustrating the effect of the proposed gradient consistency regularisation term. For easy visualisation, the hyperspectral images presented in this document have all been converted into sRGB images. This can be achieved using the method described in [?], which involves first converting the spectral data to the CIE

Table 1 PSNR results of the demosaicked images using different weighting factors for each regularisation term.

		λ_ρ=0	λ_ρ=0.01	λ_ρ=0.1	λ_ρ=1	λ_ρ=10
$\lambda_{TV} = 0$	$\lambda_{Tik} = 0$	-	36.33	36.33	36.33	36.33
$\lambda_{TV} = 0$	$\lambda_{Tik} = 0.1$	32.54	39.82	40.32	38.67	36.78
$\lambda_{TV} = 0$	$\lambda_{Tik} = 1$	32.54	36.23	39.93	40.35	38.67
$\lambda_{TV} = 0$	$\lambda_{Tik} = 10$	32.54	33.66	36.23	39.92	40.35
$\lambda_{TV} = 1e{-4}$	$\lambda_{Tik} = 0$	31.64	40.46	39.12	36.89	36.38
$\lambda_{TV} = 1e{-4}$	$\lambda_{Tik} = 0.1$	32.15	39.71	41.47	38.84	36.83
$\lambda_{TV} = 1e{-4}$	$\lambda_{Tik} = 1$	32.53	36.21	39.92	41.37	38.69
$\lambda_{TV} = 1e{-4}$	$\lambda_{Tik} = 10$	32.54	33.66	36.23	39.92	41.35
$\lambda_{TV} = 1e{-3}$	$\lambda_{Tik} = 0$	31.64	35.16	38.71	37.80	36.73
$\lambda_{TV} = 1e{-3}$	$\lambda_{Tik} = 0.1$	30.48	37.41	41.25	39.50	37.14
$\lambda_{TV} = 1e{-3}$	$\lambda_{Tik} = 1$	32.29	35.90	39.81	41.50	38.84
$\lambda_{TV} = 1e{-3}$	$\lambda_{Tik} = 10$	32.53	33.65	36.21	39.92	41.37
$\lambda_{TV} = 1e{-2}$	$\lambda_{Tik} = 0$	31.60	33.21	34.80	37.12	37.67
$\lambda_{TV} = 1e{-2}$	$\lambda_{Tik} = 0.1$	25.15	29.36	34.39	39.07	38.05
$\lambda_{TV} = 1e{-2}$	$\lambda_{Tik} = 1$	29.83	33.03	37.42	41.26	39.51
$\lambda_{TV} = 1e{-2}$	$\lambda_{Tik} = 10$	32.29	33.33	35.90	39.81	41.50

XYZ colour space, and then transforming the XYZ images to linear RGB images. Finally, gamma correction is applied to obtain the sRGB images.

It can be seen from Figure 1 that when the weighting factor of the proposed regularisation term λ_ρ is set to 0, the result from using only traditional regularisation techniques is similar to a linearly demosaicked image. The proposed regularisation term strengthens the correlation between the spatial gradient maps of different spectral bands, which results in enhanced image sharpness.

A quick way to test the performance of our proposed regularisation term without training a neural network is to solve the direct inverse problem. This can be achieved by directly finding a hyperspectral image such that the sum of all regularisation terms are minimised, as expressed in Eq. (2) in the main paper. The minimisation can be achieved by common iterative methods such as Broyden–Fletcher–Goldfarb–Shanno [**?**] algorithm and Adam optimisation.

The values of the weighting factors for all regularisation terms, including λ_{Tik}, λ_{TV} and λ_ρ, were determined by solving the direct inverse problem using one of the images from the HELICoiD dataset, and then calculating the PSNR of the results. Adam optimisation with initial learning rate of 0.01, $\beta_1 = 0.5$ and $\beta_2 = 0.99$ was used during the experiment for fast minimisation, and it took 500 iterations to obtain the demosaicking results. These results are shown in Table 1, where it can be seen that the highest PSNR can be achieved when $\lambda_{Tik} = 1$, $\lambda_{TV} = 1e{-3}$ and $\lambda_\rho = 1$ or when $\lambda_{Tik} = 10$, $\lambda_{TV} = 1e{-2}$ and $\lambda_\rho = 10$, which is just multiples of the former set of weights. Therefore, we chose $\lambda_{Tik} = 1$, $\lambda_{TV} = 1e{-3}$ and $\lambda_\rho = 1$ for all unsupervised network training.

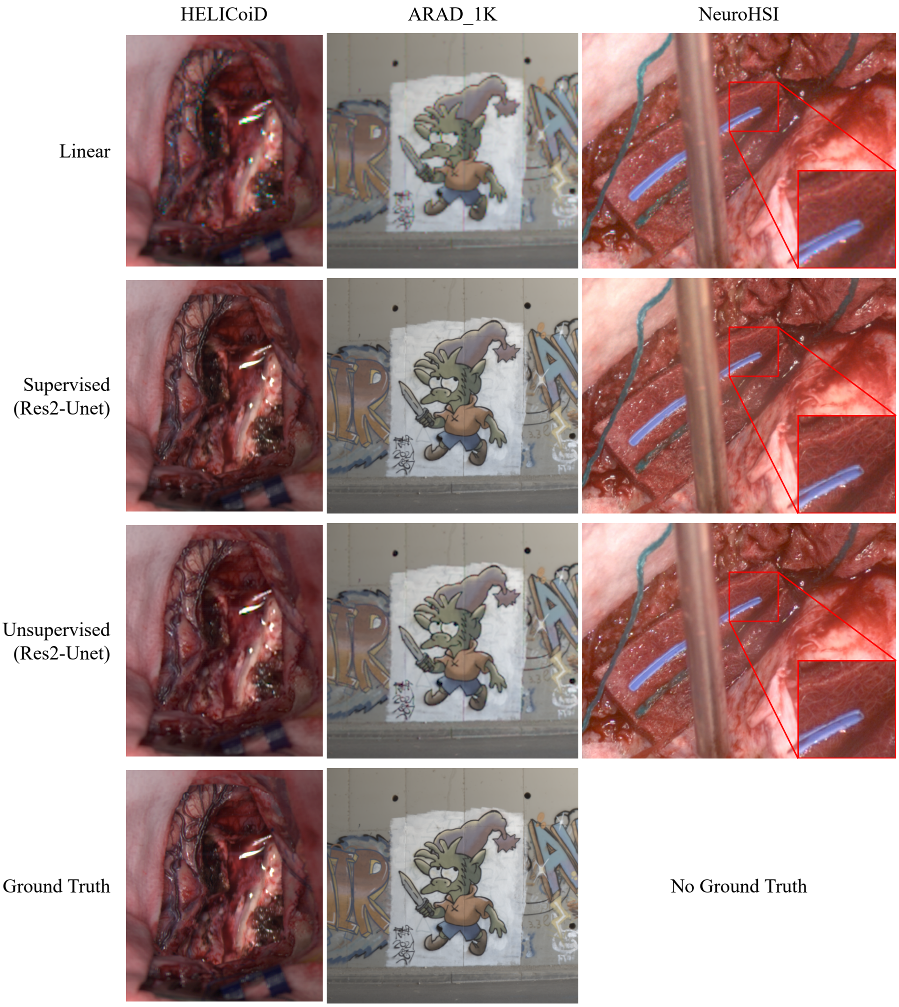

Fig. 2 Example demosaicking results from each dataset (HELICoiD, ARAD_1K, NeuroHSI).

3 Additional Examples from the Results

Figure 2 shows some additional example results of linear demosaicking, supervised and unsupervised trained Res2-Unet model, as well as the ground truths respectively on HELICoiD [?], ARAD_1K [?] and NeuroHSI datasets. Since there are no high-resolution images as ground truth for the NeuroHSI dataset, training a supervised network model on this dataset is not possible. Therefore, the supervised Res2-Unet results for NeuroHSI dataset were inferred by directly using the supervised Res2-Unet model trained from HELICoiD dataset. The reason to choose the HELICoiD-trained model rather than the ARAD_1K model is that both HELICoiD and NeuroHSI are neurosurgical

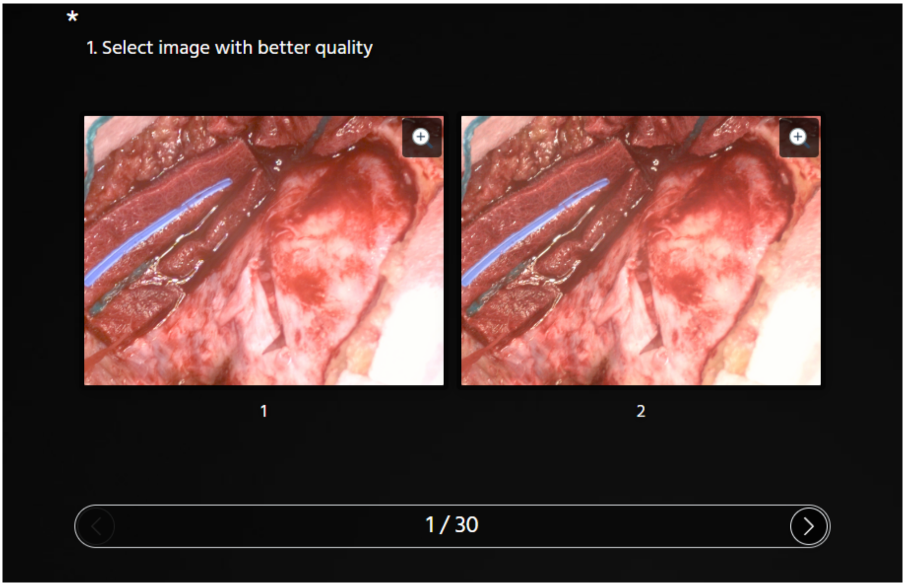

Fig. 3 The user interface of the survey. Zoom option is provided to help the participants with observing the images in more details.

image datasets, so there is less domain gap compared to ARAD_1K which are all natural scene images. Adapting ARAD_1K-trained networks on NeuroHSI images for better results may involve methods such as transfer learning, which is not the primary focus of this work.

4 Additional Information on the User Study

The aim of the user study was to rank the three demosaicking methods based on the demosaicked NeuroHSI images: linear demosaicking (L), supervised training from the HELICoiD dataset (SL) and unsupervised training from the NeuroHSI dataset (UL). We chose Res2-Unet as the network to generate both supervised and unsupervised results, because from the quantitative analysis of the results on both HELICoiD and ARAD_1K datasets, supervised Res2-Unet achieved the highest demosaicking accuracy.

[**?**] argues that forced-choice pairwise comparison is the fastest and the most accurate type of user study for image quality assessment. Therefore, we designed a two-alternative forced-choice (2AFC) image quality survey, where observers needed to compare two images at a time and choose one with better quality without giving any rating scales. Hence, the three methods were compared by directly inferred pairwise comparison: L vs SL, L vs UL, and SL vs UL. There are 30 test images in the NeuroHSI dataset, thus the 90 demosaicked results from all three methods can form 90 pairwise comparisons as the

Table 2 Results of the image quality assessment survey summarising the preferences for each pairwise comparison. The number refers to the number of votes that the demosaicking method in the row is preferred over the method in the column.

	L	SL	UL
L	-	13	10
SL	107	-	57
UL	110	63	-

survey questions. It was not practical to ask each participant to make judgements on all 90 image pairs, so we divided them into 3 separate surveys, each containing 30 image pairs.

The participants of this survey were all neurosurgical experts with 2 to 15 years of experience. Each participant was randomly assigned with one of the three surveys on a website. The introductory page provided some instructions about the recommended screen size, browsers as well as the information about the survey tools. Then the two images to compare were presented to the participants, as shown in Figure 3.

We received 4 responses for each of the 3 surveys, so there are 12 responses in total. The results are summarised in Table 2, where it can be seen that when compared with linear demosaicking, the supervised demosaicking received 89.2% of the votes, and our proposed unsupervised demosaicking received 91.7% of the votes. When directly comparing the unsupervised demosaicking images against supervised demosaicking, our proposed method still received 52.5% of the votes.

Bradley-Terry model was applied to map probability of preference to scales to describe which demosaicking methods are more preferred by the experts, as suggested by [**?**] for image quality assessment. In the Bradley-Terry model, consider K number of methods to be compared. For method i and j, denote the probability that method i wins over method j as:

$$p_{ij} = \mathbb{P}(i > j) = \frac{\pi_i}{\pi_i + \pi_j} \tag{9}$$

where π_i is the scale value indicating the preference of the method i. Let w_{ij} be the number of votes that method i is preferred over method j. Assume the vote from each pairwise comparison is independent, Bradley-Terry model describes the log-likelihood of the scale parameter $\pi = [\pi_1, ..., \pi_k], k \in [1, K]$ as

$$L(\pi) = \sum_{i}^{K} \sum_{j}^{K} [w_{ij} \log \pi_i - w_{ij} \log(\pi_i + \pi_j)] \tag{10}$$

[**?**] proposed to use MM-algorithm to find a maximum likelihood estimation of π by performing an iterative update until convergence:

$$\pi_i^{(n+1)} = \frac{\sum_{j=1}^{K} w_{ij}}{\sum_{j=1}^{K} \frac{w_{ij}+w_{ji}}{\pi_i^{(n)}+\pi_j^{(n)}}} \tag{11}$$

Fitting a Bradley-Terry model with the results of the user study in Table 2 using (11), we can get an estimated preference scale of $\pi = (0.050, 0.445, 0.505)$ for L, SL and UL respectively. This result shows that the experts consider the images recovered from our proposed demosaicking method to have similar quality as the images from a supervised model, and the baseline linear demosaicking is the least favourable method.
